# Reduced forced vital capacity is independently associated with ethnicity, metabolic factors and respiratory symptoms in a Caribbean population: a cross-sectional study

**DOI:** 10.1186/s12890-019-0823-9

**Published:** 2019-03-14

**Authors:** Sateesh Sakhamuri, Fallon Lutchmansingh, Donald Simeon, Liane Conyette, Peter Burney, Terence Seemungal

**Affiliations:** 1 Faculty of Medical Sciences, Department of Clinical Medical Sciences, The University of the West Indies, St. Augustine, Trinidad and Tobago; 2 Dean’s Office, Faculty of Medical Sciences, The University of The West Indies, St. Augustine Campus, St. Augustine, Trinidad and Tobago; 3 South-West Regional Health Authority, San Fernando, Trinidad and Tobago; 4 National Heart and Lung Institute, Imperial College, London, UK

## Abstract

**Background:**

Relationships between low forced vital capacity (FVC), and morbidity have previously been studied but there are no data available for the Caribbean population. This study assessed the association of low FVC with risk factors, health variables and socioeconomic status in a community-based study of the Trinidad and Tobago population.

**Methods:**

A cross-sectional survey was conducted using the Burden of Obstructive Lung Disease (BOLD) study protocol. Participants aged 40 years and above were selected using a two-stage stratified cluster sampling. Generalized linear models were used to examine associations between FVC and risk factors.

**Results:**

Among the 1104 participants studied a lower post-bronchodilator FVC was independently associated with a large waist circumference (− 172 ml; 95% CI, − 66 to − 278), Indo-Caribbean ethnicity (− 180 ml; 95% CI, − 90 to − 269) and being underweight (− 185 ml; 95% CI, − 40 to − 330). A higher FVC was associated with smoking cannabis (+ 155 ml; 95% CI, + 27 to + 282). Separate analyses to examine associations with health variables indicated that participants with diabetes (*p* = 0∙041), history of breathlessness (p = 0∙007), and wheeze in the past 12 months (*p* = 0∙040) also exhibited lower post-bronchodilator FVC.

**Conclusion:**

These findings suggest that low FVC in this Caribbean population is associated with ethnicity, low body mass index (BMI), large waist circumference, chronic respiratory symptoms, and diabetes.

**Electronic supplementary material:**

The online version of this article (10.1186/s12890-019-0823-9) contains supplementary material, which is available to authorized users.

## Introduction

More than one and a half centuries after Hutchinson’s design of a spirometer to determine the ‘capacity for life,’ the forced vital capacity (FVC) remains a good predictor of mortality and morbidity. It is related to all-cause mortality even in the general population [1, 2] and can predict it better than systolic blood pressure or body mass index (BMI) [3]. Studies from the developed world have also shown significant associations of FVC with cardiovascular disease [4, 5], cardiovascular events [6], sudden cardiac death [7], metabolic syndrome [8], diabetes [9, 10], and the progression of chronic kidney disease [11]. There are relatively few studies that have examined the risk factors for a low FVC though this has often been attributed to “normal” ethnic differences.

Few spirometry based studies have been conducted on the Caribbean population. These studies have focused on airway obstruction and were performed either in specialty clinics or hospital. Two of them showed low forced expiratory volume in one second (FEV1) or FVC associated with vascular disease [12, 13] and another, FVC with systemic inflammation in diabetic patients [14].

We studied FVC in a national community-based study of non-institutionalized adults aged 40 years and over and living in Trinidad and Tobago, using the Burden of Obstructive Lung Disease (BOLD) study methodology. We investigated potential risk factors as well as the relation of FVC to the health and socioeconomic status. Since the use of universal cut-offs to define abnormal spirometry is contentious [15], we have analysed FVC as a continuous variable to assess its associations, including those with age, sex and ethnicity. In addition, we also studied similar associations with pre-bronchodilator FVC; and pre and post-bronchodilator FEV1.

## Methods

### Setting

Trinidad and Tobago, a high human development indexed country in the Caribbean, has a uniquely diverse population of predominantly East Indian and African descent. More than half of the population aged 20 years or more (55.5% of males and 66.1% of females) are overweight and obese [16]. The country also possesses a high burden of diabetes and cardiovascular diseases which were determined as the top two causes of death and disability in 2016 (Data was sourced from the IHME GBD profile. http://www.healthdata.org/trinidad-and-tobago.).

### Study design

A cross-sectional survey was conducted across the 15 administrative districts of Trinidad and Tobago, a country with about 1.3 million inhabitants including 39% aged 40 years and above [17]. The study was approved by the ethics committees of the Faculty of Medical Sciences of the University of the West Indies and the Ministry of Health, Trinidad and Tobago.

After obtaining consent, participants aged 40 years and above were asked to answer a core questionnaire focusing on respiratory symptoms, health status, activity limitation, use of healthcare services, and exposure to potential risk factors, such as cigarette smoke. The participants also performed spirometry if there were no contraindications for forced expiratory manoeuvres. Additional questionnaires on indoor air pollution and occupational exposures were administered before the post-bronchodilator spirometry manoeuvres. A wealth score, using a Mokken scale [18] was applied to differentiate the socio-economic status of individual participants. This score was calculated based on the ownership of 10 household assets.

### Spirometry

Spirometry was performed according to the 1994 American Thoracic Society (ATS) criteria [19], using the Easy-One portable spirometer (ndd Medizintechnik; Zurich, Switzerland), with the participant in a seated position and pre and post-bronchodilator spirometry (15 min after administering 200 μg salbutamol via metered-dose inhaler with a valve spacer) performed following the BOLD methodology [20]. The difference between the largest and second largest FEV1 and FVC values of < 200 ml was considered as reproducible [20]. A plateau for at least one second after an exhalation time of at least 6 s was considered as a valid end-of-test criterion [19]. Spirometry data were transmitted electronically to the BOLD pulmonary function reading centre in London, where each spirogram was reviewed. A good spirometry had to meet ATS criteria for acceptability, including having at least three attempts, two of which were acceptable [21]. Spirometry technicians were continuously monitored and whenever their quality scores dropped below a pre-set level, they were asked to stop testing, and undergo retraining and recertification. Among the acceptable efforts, the best post-bronchodilator FEV1 and FVC values, even if they were from different curves were used for statistical analyses [19].

### Sampling

Participants were selected using two-stage stratified cluster sampling. The study was based on the BOLD protocol that required a minimal sample size of 600 persons above the age of 40 years. The actual sample size, inflated to take into account an expected rate of non-response and unacceptable spirometry (20%) and the clustered nature of the sampling, was 1209 households. A total of 1469 eligible participants were identified from these households and invited to participate.

### Statistical analyses

Chi-square tests were used to examine differences in categorical variables and Student’s t-test to examine differences in continuous variables. We checked for differences between responders and non-responders and between those with and without acceptable spirometry. Complex Samples General Linear Models (SPSS Version 25) were used to study associations between FVC and the risk factors. This enabled the application of the stratified cluster sampling structure of the data in the analysis. Weights were also used in the analyses. Base weights were calculated as the inverse of the probability of each participant’s selection. Final weights were determined by adjusting for the age and gender distribution of the national population, using census data.

Age, sex, height, and height-squared are strong predictors of lung function [22] and as these four variables accounted for 60.5% of FVC variance, they were entered as covariates in all analyses. Age squared was not a significant predictor in our analyses and was not used as a covariate. Separate analyses were conducted for each risk factor. All the risk factors that were significantly associated with FVC were subsequently entered in a final model to determine independent predictors. We also used General Linear Models to conduct separate regression analyses to examine associations between FVC and the various health status indicators, and respiratory symptoms. The Complex Samples Analysis module was also used to estimate the prevalence and 95% CI for chronic airflow obstruction.

## Results

**Fig. 1:**
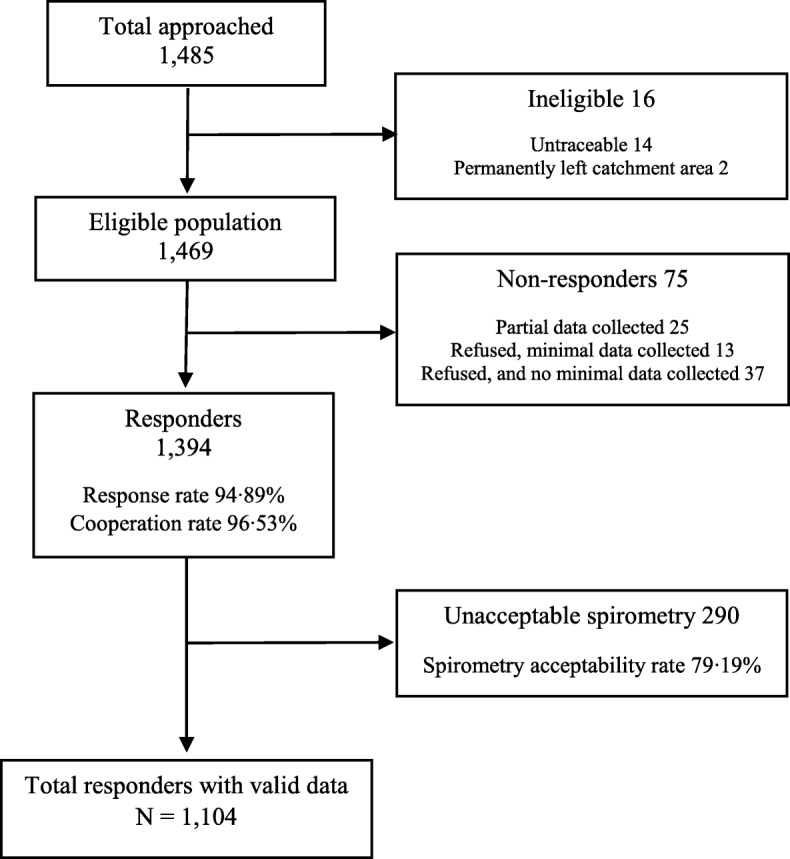
Sampling of participants in the BOLD-Trinidad and Tobago study

Out of a total eligible sample of 1469 individuals, 1394 completed the core questionnaire and undertook spirometry. Among them, 1104 successfully performed spirometry, as per the BOLD study quality control criteria (Fig. 1). Of the individuals approached 95% responded (95% response rate) and of these 97% agreed to participate (97% co-operation rate). Spirometry acceptability rate was 79%. Younger participants, those of Indo-Caribbean descent and those who had no chronic respiratory symptoms had higher rates of acceptable spirometry (*p* < 0.005 in all cases) (Additional file 1: Table S1). Smoking status, BMI and the presence of doctor-diagnosed respiratory disease did not show association with the participants’ spirometry acceptability.

The majority of participants were females (60%), and the sample’s age and ethnic distributions matched well with the recent national census data [17]. Overall, the sample comprised mainly persons of Asian or African ancestry (78%), with secondary or higher level education (53%), who were overweight or obese (70%), and who were exposed to indoor air pollutants (55%) (Table 1). Mean BMI and waist circumferences were higher among Afro-Caribbeans than Indo-Caribbeans (29.59 kg/m^2^ vs. 27.90 kg/m^2^; 97.71 cm vs. 95.71 cm, respectively; *p* < 0.03 in all cases). 27% of the participants gave a history of smoking, which was four times more prevalent in males than females. Among the smokers, more than half were current smokers and one third had also smoked cannabis. 85% of participants had ownership of eight or more of the household amenities in the inventory.

**Table 1:** Demographics, anthropometry, smoking history and indoor air pollutant exposure of the BOLD Trinidad and Tobago study participants

Variable	Male (443)	Female (661)	Total (1104)
Age in years			
40–49	152 (34.3%)	287 (43.4%)	439 (39.8%)
50–59	145 (32.7%)	193 (29.2%)	338 (30.6%)
60–69	90(20.3%)	117 (17.7%)	207 (18.8%)
70+	56 (12.6%)	64 (9.7%)	120 (10.9%)
Ethnicity			
Indo-Caribbean	191 (43.1%)	269 (40.7%)	460 (41.7%)
Afro-Caribbean	169 (38.1%)	233 (35.2%)	402 (36.4%)
Mixed/ other	83 (18.7%)	159 (24.1%)	242 (21.9%)
Highest completed level of education			
Primary /none	205 (46.8%)	314 (47.5%)	521 (47.2%)
Secondary	134 (30.2%)	216 (32.7%)	350 (31.7%)
Vocational	79 (17.8%)	90 (13.6%)	169 (15.3%)
University	23 (5.2%)	41 (6.2%)	64 (5.8%)
Employment status			
Employed	287 (64.8%)	328 (49.6%)	615 (55.7%)
Not working	17 (3.5%)	23 (3.5%)	40 (3.6%)
House-person	7 (1.6%)	208 (31.5%)	215 (19.5%)
Retired	122 (27.5%)	88 (13.3%)	210 (19.0%)
Other	10 (2.3%)	14 (2.1%)	24 (2.2%)
Wealth score (Mean (SD))	8.85 (1.62)	9.03 (1.31)	8.96 (1.44)
BMI groups			
Underweight (< 18.5 kg/m^2^)	11 (2.5%)	15 (2.3%)	26 (2.4%)
Normal (18.5–24.9 kg/m^2^)	162 (36.6%)	146 (22.1%)	308 (27.9%)
Overweight (25–29.9 kg/m^2^)	174 (39.3%)	207 (31.3%)	381 (34.5%)
Obese (≥30 kg/m^2^)	96 (21.7%)	293 (44.3%)	389 (35.2%)
Waist circumference			
Normal	300 (67.7%)	162 (24.5%)	461 (41.8%)
Abnormal (≥102 cm for males, ≥88 cm for females)	143 (32.3%)	499 (75.4%)	642 (58.2%)
Waist Hip ratio			
Normal	152 (34.4%)	205 (31.0%)	357 (32.4%)
Abnormal (> 0.9 for males, > 0.85 for females)	290 (65.6%)	456 (68.9%)	746 (67.6%)
Smoking status			
Current	121 (27.3%)	36 (5.4%)	157 (14.2%)
Former	104 (23.5%)	41 (6.2%)	145 (13.1%)
Never	218 (49.2%)	584 (88.4%)	802 (72.6%)
Pack-year categories			
Never	219 (49.5%)	584 (88.4%)	803 (72.8%)
0–10	67 (15.2%)	35 (5.3%)	102 (9.2%)
10–20	56 (12.7%)	21 (3.2%)	77 (7.0%)
20 +	100 (22.6%)	21 (3.2%)	121 (11.0%)
Ever smoked cannabis	72 (16.3%)	24 (3.6%)	96 (8.7%)
Exposure to second hand smoke	152 (34.3%)	220 (33.3%)	372 (33.7%)
Working in a dusty environment for > 1 year	238 (53.7%)	161 (24.4%)	399 (36.1%)
Indoor open fire with coal used for cooking	87 (19.9%)	99 (15.1%)	186 (17.0%)
Indoor open fire with wood used for cooking	188 (42.9%)	249 (37.9%)	437 (39.9%)
Kerosene used for cooking	163 (37.2%)	249 (37.9%)	412 (37.6%)
Indoor air pollutant exposure: coal, wood or kerosene			
Exposure to one	126 (28.4%)	179 (27.1%)	305 (27.6%)
Exposure to two	75 (16.9%)	125 (18.9%)	200 (18.1%)
Exposure to all three	54 (12.2%)	56 (8.5%)	110 (10.0%)
None	188 (42.4%)	301 (45.5%)	489 (44.3%)

Data are presented as n (%) if not stated otherwise

About one-third of the study participants mentioned at least one of the four symptoms - cough, phlegm, wheeze, and breathlessness in the past 12 months. Also, nearly 10% reported a doctor diagnosed respiratory disease (Table 2). 37% had at least one known co-morbidity, the most prevalent conditions being hypertension (28%) and diabetes (15%). Indo-Caribbeans had a higher diabetes prevalence than the Afro-Caribbeans and Mixed/ other ethnic groups (21, 10, and 12% respectively). This is the only health variable observed to be different between the ethnic groups. Gender differences in health status were noted in breathlessness, (*p* < 0.001) and doctor-diagnosed respiratory diseases (*p* = 0.03). In each case, the rates were higher in women than in men (Table 2).

**Table 2:** Health variables of BOLD Trinidad and Tobago study participants

Variable	Male (443)	Female (661)	Total (1104)
Chronic cough	30 (6.8%)	52 (7.9%)	82 (7.4%)
Chronic phlegm	13 (2.9%)	27 (4.1%)	40 (3.6%)
Wheezing in last 12 months	44 (9.9%)	85 (12.9%)	129 (11.7%)
Breathlessness	54 (12.5%)	136 (21.7%)	190 (17.9%)
Symptomatic (any single respiratory symptom)	134 (30.2%)	248 (37.5%)	382 (34.6%)
Self-reported chronic bronchitis	5 (1.1%)	11 (1.7%)	16 (1.4%)
Doctor diagnosed COPD, chronic bronchitis or emphysema	3 (0.7%)	14 (2.1%)	17 (1.5%)
Doctor diagnosed asthma	34 (7.7%)	75 (11.3%)	109 (9.9%)
Doctor diagnosed respiratory disease	35 (7.9%)	79 (12.0%)	114 (10.3%)
Doctor diagnosed any other medical condition	146 (33.0%)	255 (38.6%)	401 (36.3%)
Doctor diagnosed heart disease	27 (6.1%)	33 (5.0%)	60 (5.4%)
Heart failure	12 (2.7%)	10 (1.5%)	22 (2.0%)
Hypertension	112 (25.3%)	202 (30.6%)	314 (28.4%)
Diabetes	59 (13.3%)	109 (16.5%)	168 (15.2%)
Stroke	5 (1.1%)	4 (0.6%)	9 (0.8%)
Lung cancer	0 (0%)	1 (0.2%)	1 (0.1%)
Tuberculosis	0 (0%)	0 (0%)	0 (0%)
Presence of any single comorbidity	147 (33.2%)	257 (38.9%)	404 (36.6%)
Hospitalised as a child for breathing problems prior age 10	6 (1.4%)	10 (1.5%)	16 (1.5%)

Data are presented as n (%)

### Risk factors for low FVC

FVC values were higher in men than women (mean difference = 1070 ml; 95%CI = 991, 1148; *p* < 0.001). These values were also positively correlated with height (b = 0.052; 95%CI = 0.047, 0.056; *p* < 0.001) and negatively associated with age (b = − 0.026; 95%CI = − 0.031, − 0.021; *p* < 0.001).

**Fig. 2:**
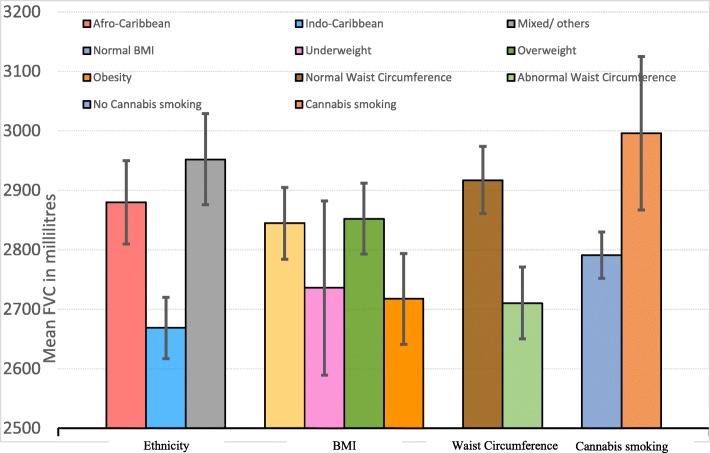
Mean post-bronchodilator FVCs adjusted for age, sex, height and height square among various groups that are statistically significant (*p* < 0.05). Bars represent the mean FVC in millilitres and error bars the 95% CI

The mean FVC and FEV1 values adjusted for age, sex, height, and height-squared are tabulated in Table 3 by the potential risk factors. There were significant post-bronchodilator FVC differences by ethnicity (*p* < 0.001), BMI group (*p* = 0.024), abnormal waist circumference (*p* < 0.001), abnormal waist-hip-ratio (*p* < 0.001), and whether they smoked cannabis (*p* = 0.004). Indo-Caribbeans showed lower mean FVCs than Afro-Caribbeans and other ethnic groups (Table 3 and Fig. 2). BMI presented a non-linear relation with low FVC. Underweight and obese subjects displayed lower FVCs than those with normal body habitus and overweight people. People with central obesity (abnormal waist circumference and waist-hip ratio) also showed lower FVCs. On the other hand, smokers of cannabis had higher FVC scores than persons who never smoked cannabis. Cigarette smoking status, history of pack-years, second-hand smoking, childhood exposure to smoking, indoor air pollutant exposure, and working in a dusty environment for more than 1 year were not associated with FVC values.

**Table 3:** Mean adjusted^a^ pre and post-bronchodilator (BD) forced expiratory volume in one second (FEV1) and forced vital capacity (FVC) values (in ml) by the various potential risk factors

Variable	Adjusted Pre-BD Mean FEV1	Adjusted Post-BD Mean FEV1	Adjusted Pre-BD Mean FVC	Adjusted Post-BD Mean FVC
Ethnicity	***	***	***	***
Indo-Caribbean	2085	2133	2661	2669
Afro-Caribbean	2212	2268	2853	2880
Mixed/ other	2284	2331	2951	2952
BMI group			*	*
Underweight (< 18.5 kg/m^2^)	2098	2146	2739	2736
Normal (18.5–24.9 kg/m^2^)	2198	2239	2859	2845
Overweight (25–29.9 kg/m^2^)	2200	2257	2821	2852
Obese (≥30 kg/m^2^)	2119	2175	2693	2718
Waist circumference	***	***	***	***
Normal	2261	2300	2923	2917
Abnormal^b^	2094	2158	2674	2710
Waist Hip ratio	***	***	***	***
Normal	2263	2308	2928	2919
Abnormal^c^	2126	2182	2721	2748
Smoking status				
Current smoker	2157	2242	2834	2894
Ex-smoker	2158	2196	2757	2769
Never smoker	2176	2223	2784	2791
Smoking pack years				
Never	2176	2223	2783	2790
0–10	2217	2247	2809	2819
10–20	2152	2232	2795	2824
20+	2109	2192	2787	2852
Smoking and respiratory symptoms		*		
Never smoker with no symptoms	2201	2257	2812	2819
Never smoker with symptoms	2119	2148	2720	2725
Ever smoker with no symptoms	2211	2261	2859	2876
Ever smoker with symptoms	2087	2173	2717	2780
Ever smoked cannabis			*	**
No	2167	2220	2777	2791
Yes	2255	2302	2984	2996
Second-hand smoking				
No	2176	2234	2773	2817
Yes	2160	2200	2796	2777
Indoor air pollutant exposure (coal, wood or kerosene)	*	*	*	
None	2203	2249	2838	2846
Exposure to one	2119	2188	2712	2742
Exposure to two	2223	2266	2821	2831
Exposure to all three	2089	2132	2735	2753
Worked in a dusty environment for > 1 year				
No	2183	2227	2780	2784
Yes	2150	2216	2803	2835
Smoking exposure during childhood	*			
No	2207	2250	2801	2818
Yes	2144	2203	2772	2794
Highest level of education	***	*	***	
Primary/none	2137	2197	2747	2775
Secondary	2159	2212	2778	2805
Vocational	2228	2259	2847	2819
University	2363	2397	3027	2974
Education – years of schooling	*			
0–6 years	2073	2162	2680	2730
7+ years	2182	2230	2801	2811
Current employment status				
Employed	2195	2240	2805	2815
Not working	2152	2237	2788	2880
House person	2140	2215	2717	2753
Retired	2111	2164	2772	2775
Other	2361	2362	3082	3024

Significant *p*-values are shown and denoted by * < 0.05; ** < 0.005; *** < 0.001

^a^Adjusted for age, sex, height and height-squared with covariates were fixed at the following values: Sex = 0.49; height = 166.43; height-squared = 27,818.0145; Age = 54.93

^b^Abnormal waist circumference: ≥102 cm for males and ≥ 88 cm for females

^c^Abnormal waist hip ratio: > 0.9 for males, > 0.85 for females

Multiple regression analysis of the risk factors that were significant after adjusting for age, sex, height, and height-squared indicated that post-bronchodilator FVC was lower in those with increased waist circumference (− 172 ml), Indo-Caribbean participants (− 180 ml) and those who were underweight (− 185 ml), and higher in those who smoked cannabis (+ 155 ml) (Table 4).

**Table 4:** Results of the general linear models analyses for the significant risk factors for post-bronchodilator forced vital capacity (FVC)

Variables	Categories	Models with Individual Risk Factors^a^			Multivariate Model^a^			p-values (Multivariate model)
		Coefficient (ml)	95% CI		Coefficient (ml)	95% CI		
Ethnicity	Afro-Caribbean	Baseline						< 0.001
	Indo-Caribbean	−211	−302	−120	−180	−269	−90	
	Mixed/Other	73	−35	180	79	−27	185	
BMI^b^	Normal	Baseline						0.01
	Underweight	−109	−261	44	−185	−330	−40	
	Overweight	8	−77	93	68	−24	161	
	Obese	− 127	−228	−26	−15	−128	98	
Abnormal waist circumference^c^	Yes	−207	− 296	−119	−172	−278	−66	< 0.001
Abnormal waist–hip ratio ^d^	Yes	− 170	− 246	−95	−71	−145	2	0.057
Ever smoked cannabis	Yes	205	67	342	155	27	282	0.018

^a^All models included sex, age, height and height-squared. ^b^Normal BMI = 18.5–25.0 Kg/m^2^; Underweight BMI < 18.5 Kg/m^2^; Overweight BMI = 25.0–29.9 Kg/m^2^; Obese BMI ≥30 Kg/m^2^. ^c^**:** Abnormal waist circumference ≥ 102 cm for males and ≥ 88 cm for females. ^d^: Abnormal wait-hip ratio ≥ 0.90 for males and ≥ 0.85 for females

Risk factors for low pre-bronchodilator FVC were of similar significance to those for post-bronchodilator FVC except that indoor air pollution and levels of education were related to pre-bronchodilator FVC but not to post-bronchodilator FVC (Tables 3, 4 and Additional file 1: Table S2).

### FVC and health variables

The mean adjusted FVC and FEV1 scores by the various symptoms and health status variables are listed in Table 5. Participants with known diabetes (*p* = 0.041), with a history of breathlessness (*p* = 0.007), and wheeze in the past 12 months (*p* = 0.040) exhibited lower FVC. Diagnosed respiratory disease, hypertension, cardiac disease, history of cough or phlegm, hospitalization before the age of 10 years, and family history of airway disease were not associated with FVC.

**Table 5:** Mean adjusted^a^ pre and post bronchodilator (BD) forced expiratory volume in one second (FEV1) and forced vital capacity (FVC) values (in ml) by the various health variables

Variable	Adjusted Pre-BD Mean FEV1	Adjusted Post-BD Mean FEV1	Adjusted Pre-BD Mean FVC	Adjusted Post-BD Mean FVC
Hospitalisations prior to the age 10			*	
No	2171	2223	2786	2801
Yes	2246	2244	3042	3032
Known asthma	***	***		
No	2192	2242	2800	2812
Yes	1967	2049	2675	2727
Known respiratory disease	***	***	*	
No	2195	2244	2803	2815
Yes	1955	2037	2654	2707
Known hypertension				
No	2153	2197	2806	2820
Yes	2177	2233	2741	2764
Known diabetes			*	*
No	2127	2166	2802	2818
Yes	2179	2233	2709	2727
Known cardiac disease				
No	2084	2132	2795	2813
Yes	2176	2228	2673	2662
Presence of any known comorbidity			*	
No	2184	2236	2814	2824
Yes	2146	2199	2742	2770
Chronic cough				
No	2173	2226	2784	2799
Yes	2133	2182	2807	2827
Phlegm				
No	2172	2223	2792	2806
Yes	2143	2208	2755	2783
Wheeze in the last 12 months	***	***	***	*
No	2200	2247	2815	2821
Yes	1946	2041	2579	2681
Breathlessness	**	**	***	*
No	2216	2270	2837	2850
Yes	2071	2121	2678	2708
Family history of airway disease				
No	2167	2220	2782	2799
Yes	2272	2281	2928	2919

Significant *p*-values are shown and denoted by * < 0.05; ** < 0.005; *** < 0.001

^a^Adjusted for age, sex, height and height-squared with covariates were fixed at the following values: Sex = 0.49; height = 166.43; height-squared = 27,818.0145; Age = 54.93

### Risk factors for low FEV1

Low post-bronchodilator FEV1 was also independently associated with Indo-Caribbean ethnicity (− 125 ml) and abnormal waist circumference (− 108 ml) (Additional file 1: Table S4). In contrast to FVC, low FEV1 showed an independent association with indoor air pollutant exposure (− 95 ml for all three exposures) but did not show a relation with BMI and cannabis smoking. Further, pre-bronchodilator FEV1 showed associations with abnormal waist-hip ratio (− 69 ml) and highest level of education (+ 168 ml for university education).

## Discussion

To our knowledge this is the first published study of lung function in the general population of a Caribbean country and provides new information on the associations of FVC with participant demographics, socio-economic status and morbidity. We found lower FVCs among the Indo-Caribbean population, those with a low BMI and with central obesity. Individuals with a low FVC had more respiratory symptoms.

We observed low FVCs among Indo-Caribbeans compared to Afro-Caribbeans in our study by abut 8% despite the similar prevalence of abnormal waist circumference (57.0% vs. 58.7%; *p* = 0.751) and a lower prevalence of obesity (30.0% vs. 41.8%; *p* = 0.008), (Table 6). The lower volumes among Indo-Caribbeans compared with the population of African descendant were consistent with the results from Global differences in lung function by region Prospective Urban Rural Epidemiology (PURE) study [23]. This contrasts with the recently published Canadian Health Measures Survey reference values [24] which showed higher FVCs among those of South Asian compared with those of African descent.

**Table 6:** Risk Factors by Ethnicity: Afro-Caribbean (*n* = 402) vs. Indo-Caribbean (*n* = 460) vs. Mixed/Others (*n* = 242)

Variable	Afro-Caribbean	Indo-Caribbean	Mixed/ Others	p-value
Gender				0∙110
Male	169 (42.0%)	191 (41.5%)	83 (34.3%)	
Female	233 (58∙0%)	269 (58.5%)	159 (65.7%)	
Age group				0.076
40–49	145 (36.1%)	181 (39.3%)	113 (46.7%)	
50–59	134 (33.3%)	136 (29.6%)	68 (28.1%)	
60–69	80 (19.9%)	95 (20.7%)	32 (13.2%)	
70+	43 (10.7%)	48 (10.4%)	29 (12.0%)	
BMI group^a^				0.008
Underweight	7 (1.7%)	10 (2.2%)	9 (3.7%)	
Normal	93 (23.1%)	145 (31.5%)	70 (28.9%)	
Overweight	134 (33.3%)	167 (36.3%)	80 (33.1%)	
Obesity	168 (41.8%)	138 (30.0%	83 (34.3%)	
Waist circumference^b^				0.751
Abnormal	236 (58.7%)	262 (57.0%)	144 (59.8%)	
Waist-Hip ratio^c^				< 0.001
Abnormal	238 (59.2%)	352 (76.5%)	156 (64.7%)	
Smoking status				0.240
Current	56 (13.9%)	61 (13.3%)	40 (16.5%)	
Ex	54 (13.4%)	52 (11.3%)	39 (16.1%)	
Never	292 (72.6%)	347 (75.4%)	163 (67.4%)	
Smoking pack years				0.244
Never	293 (72.9%)	347 (75.6%)	163 (67.4%)	
0–10	35 (8.7%)	41 (8.9%)	26 (10.7%)	
10–20	25 (6.2%)	28 (6.1%)	24 (9.9%)	
20+	49 (12.2%)	43 (9.4%)	29 (12.0%)	
Ever smoked Cannabis				< 0.001
Yes	49 (12.6%)	19 (4.2%)	28 (11.7%)	
Exposure to second-hand smoke				0∙001
Yes	112 (27.9%)	184 (40.0%)	76 (31.4%)	
Indoor air pollutant exposure				< 0.001
Yes	198 (49.2%)	310 (67.3%)	107 (44.2%)	
Worked in dusty environment > 1 year				0.001
Yes	174 (43.3%)	153 (33.3%)	72 (29.8%)	
Smoking exposure during childhood				0∙740
Yes	234 (58.2%)	261 (56.8%)	146 (60.3%)	
Have respiratory symptoms				0.335
Yes	128 (31.8%)	165 (35.8%)	89 (36.7%)	
Highest level of education				< 0.001
Primary / None	190 (47.3%)	243 (52.8%)	88 (36.4%)	
Secondary	113 (28.1%)	144 (31.3%)	93 (38.4%)	
Vocational	72 (17.9%)	54 (11.7%)	43 (17.8%)	
University	27 (6.7%)	19 (4.1%)	18 (7.4%)	
Years of schooling				0.200
7 or more	368 (91.5%)	405 (88.0%)	220 (90.9%)	
Current employment status				< 0.001
Employed	241 (60.0%)	241 (52.4%)	133 (55.0%)	
Not working	16 (4.0%)	9 (2.0%)	15 (6.2%)	
House person	34 (8.5%)	139 (30.2%)	42 (17.4%)	
Retired	95 (23.6%)	69 (15.0%)	46 (19.0%)	
Other	16 (4.0%)	2 (0.4%)	6 (2.5%)	

Data are presented as n (%). *BMI* body mass index. ^a^Normal BMI = 18.5–25.0 Kg/m^2^; Underweight BMI < 18.5 Kg/m^2^; Overweight BMI = 25.0–29.9 Kg/m^2^; Obese BMI ≥30 Kg/m^2^. ^b^Abnormal waist circumference: ≥ 102 cm for males and ≥ 88 cm for females. ^c^: Abnormal wait-hip ratio ≥ 0.90 for males and ≥ 0.85 for females

FVC in our population showed a nonlinear relation with BMI, comprising low volumes among those with both low and high BMI. Obesity and abnormal waist circumference related reduction in vital capacity can be explained by restriction of inspiration. Obesity-associated reduction in FVC has been observed in many studies and has been attributed to an increased impedance of the chest wall [25–27]. Studies have also shown that a 1 cm increment in waist circumference can decrease FVC by 13 ml [28]. Waist circumference is considered as a superior indicator of intra-abdominal fat [29] and may be a good gauge of its effect on diaphragm function and other ventilatory mechanics. When we adjusted FVC measures for both BMI and waist circumference the association of low FVC with a high BMI disappeared and that with waist circumference was essentially unchanged, suggesting that the link between a low FVC and a high BMI is mediated largely through mechanical effects of an increase in intra-abdominal fat. The association of a low FVC with a low BMI, however, was strengthened in the adjusted model, suggesting a more direct association. Low vital capacities have also been reported to be associated with low birth weight [30], though we have no estimate of birth weight in this population.

An increased FVC among cannabis smokers has also been reported in previous studies [31–33]. The exact cause for this increase is unclear but could reflect a “healthy smoker” effect, those with poor lung function being less likely to take up smoking cannabis. The effect of cannabis on FVC and the lack of association with FEV1 could be explained by training effects on the respiratory muscles with the habitual deep inhalations during cannabis smoking, and the likely acute bronchodilatory effects of delta-9-tetrahydrocannabinol (THC) [34]. These findings warrant careful interpretation given the potential adverse public health implications of long-term cannabis use including emphysematous bullae [35] and a twofold increased odds of obstructive lung disease [32]. Apart from cigarette smoking, the statistically nonsignificant associations with environmental factors such as exposure to indoor air pollution or solid fuel and working in a dusty environment on FVC have been observed in other studies as well [36].

We found that participants who had a low FVC had a history of wheezing or shortness of breath. This relationship has been published in previous studies [37, 38]. A low FVC was also associated with comorbidities especially diabetes. Earlier studies have found that individuals in the lowest quartile for FVC are more likely to develop insulin resistance [8] and diabetes [9] over time. A meta-analysis of 40 publications has shown a significantly lower FVC and FEV1 with preserved FEV1/FVC ratio among diabetic patients [39].

Although low socioeconomic status and poor education have been associated with reduced ventilatory function and chronic lung disease, this was not found in the current study. This may be due to either high per capita gross domestic product (GDP US$ 17,879 in 2015) with minor economic inequalities (GINI index 40.3 in 2010) among the local community (Data was sourced from the IMF press release no. 17/423. http://www.imf.org/en/News/Articles/2017/11/06/pr17423-imf-executive-board-concludes-article-iv-consultation-with-trinidad-and-tobago) compared to other developing countries or ineffectiveness of the tools used to distinguish the economic variations in this population. Although the wealth scale that we used has been shown to have good reliability [18] and has been associated with educational attainment, the majority of the sample possessed eight or more out of ten household amenities. This was similar to the situation seen in wealthy countries like Saudi Arabia [40]. The scale may need customization.

Limitations of the current study include the cross-sectional nature of the research, reliance on self-reported data and limited tools to measure the socioeconomic variations in the local population. However, there were many strengths such as our high response and cooperation rates. The diverse and evenly distributed ethnic distribution in the population, which was reflected in the sample, allowed for the examination of ethnic differences. Other strengths included the application of robust BOLD methodology, sound participant sampling, and quality assured spirometry. Most importantly we avoided the arbitrary use of ‘normal’ values for lung function assessment.

## Conclusions

Low FVC was associated with ethnicity, central obesity, chronic respiratory symptoms, and comorbidities like diabetes. Longitudinal studies are required to estimate the mortality and morbidity risk with diminished FVCs and also to compare the health effects of reduced FVC compared to reduced static lung volumes. Identifying individuals with low FVC may have clinical and public health importance and a better understanding of this condition and its origins is needed.

## Additional file

Additional file 1: Supplementary material. (DOCX 49.6 kb)

## Abbreviations

ATS: American thoracic society
BMI: Body mass index
BOLD: Burden of obstructive lung disease
FEV1: Forced expiratory volume in one second
FVC: Forced vital capacity
GDP: Gross domestic product
PURE: Prospective urban rural epidemiology study
THC: Tetrahydrocannabinol
