# Supplementary material for: Reduced forced vital capacity is independently associated with ethnicity, metabolic factors and respiratory symptoms in a Caribbean population: a cross-sectional study
Source: BMC Pulm Med. 2019 Mar 14;19:62. doi: 10.1186/s12890-019-0823-9 (PMC6416949; doi:10.1186/s12890-019-0823-9)
Supplement: Supplementary file 1 — Supplementary material. (DOCX 49.6 kb) [file 12890_2019_823_MOESM1_ESM.docx]

**Supplementary Material**

**Supplementary Definitions**

Body mass index (BMI) was calculated as the ratio between body weight in kilograms and squared height in meters. Based on National Institute of Health (NIH)^1^ and World Health Organization (WHO)^2^ guidelines BMI of <18.5 kg/m^2^; 18.5-24.9 kg/m^2^; 25-29.9 kg/m^2^ and 30 kg/m^2^ or more categories have been classified as underweight, normal, overweight, and obese, respectively. A waist circumference of ≥40 in (102 cm) for men and ≥35 in (88 cm) for women, and a waist-hip ratio of >0.9 for males, >0.85 for females were considered as abnormal (http://apps.who.int).^3^

A pack of cigarettes was taken as 20 cigarettes. A person who smoked 20 packs of cigarettes in a lifetime or more than one cigarette per day for a year was labelled as an ever-smoker as per the BOLD study methodology. Pack-years of cigarettes smoked were defined as the product of an average number of packs smoked per day and duration of smoking in years. Use of fuels such as wood, coal, and kerosene for cooking or heating was considered as evidence of exposure to significant “indoor air pollution”.^4^

Chronic bronchitis was determined by the occurrence of a persistent cough and phlegm on most days for at least three consecutive months for more than two successive years. Doctor-diagnosed chronic obstructive pulmonary disease (COPD) was self-reported and included a physician diagnosis of chronic bronchitis, emphysema, or COPD.

Response rate was defined as the ratio between number of responders and number of eligible individuals contacted. Cooperation rate was defined as the number of responders divided by the total number of responders plus active refusers.^5^

**Supplementary Data on Sampling**

Sampling process was guided by the country’s Central Statistical Office (CSO), the government agency responsible for national censuses as well as for the conduct of national labour force and other surveys. The CSO has divided the country into 15 administrative areas, which were used as the strata for the present study. Each administrative area was divided into clusters of households or Enumeration Districts (EDs). At the first stage of selection, EDs were selected with probability proportional to size. At the second stage, households were selected using systematic random sampling. No more than two eligible persons from each selected household were then invited to participate and informed consent sought.

**Supplementary Results**

There was no difference in the demographic variables between the responders and non-responders (e-Table 2). Among the responders, more acceptable spirometry was observed in the younger participants, Indo-Caribbeans, and those who had no respiratory symptoms (p<0.02 in all cases). Neither smoking status, BMI, nor doctor-diagnosed respiratory disease were associated with the participants’ spirometry performance.

Among the studied population gender differences were noted in age (p=0.02), employment status (p<0.001), wealth score (p=0.04), BMI status (p<0.001), cigarette smoking history (p<0.001), pack-years smoked (p<0.001), cannabis smoking (p<0.001), history of working in a dusty environment (p<0.001), and exposure to indoor coal for cooking (p=0.04). Males were older, less likely to be employed as a House-person or to be obese, had fewer possessions, and were more likely to be smokers of cigarettes and of cannabis, work in a dusty environment, and be exposed to indoor cooking using coal (table 1).

**References for the Supplementary Data**

1. Clinical Guidelines on the Identification, Evaluation, and Treatment of Overweight and Obesity in Adults-The Evidence Report. National Institutes of Health. *Obes Res*. 1998 Sep;6 Suppl 2:51S–209S.
2. Obesity: preventing and managing the global epidemic. Report of a WHO consultation. *World Health Organ Tech Rep Ser*. 2000;894:i–xii, 1-253.
3. Waist circumference and waist-hip ratio. Report of a WHO expert consultation. Geneva 8-11 December 2008. [cited 2017 Nov 25]Available from: http://apps.who.int/iris/bitstream/10665/44583/1/9789241501491_eng.pdf
4. WHO | Indoor air quality guidelines: household fuel combustion [Internet]. WHO. [cited 2018 Feb 8]. Available from: http://www.who.int/indoorair/publications/household-fuel-combustion/en/
5. Buist AS, McBurnie MA, Vollmer WM et al. International variation in the prevalence of COPD (the BOLD Study): a population-based prevalence study. *Lancet*. 2007 Sep 1;370(9589):741–50.

TABLE S1: Acceptable Spirometry (n=1104) versus Unacceptable Spirometry (n=290), BOLD-TT Study

| **Variable** | **Acceptable**  **Spirometry** | **Unacceptable**  **Spirometry** | **p-value** |
| --- | --- | --- | --- |
|  | | | |
| **Gender**  Male  Female | 443 (40∙1%)  661 (59∙9%) | 127 (43∙8%)  163 (56∙2%) | 0∙258 |
| **Age group**  40-49  50-59  60-69  70+ | 439 (39∙8%)  338 (30∙6%)  207 (18∙8%)  120 (10∙9%) | 78 (26∙9%)  78 (26∙9%)  72 (24∙8%)  62 (21∙4%) | <0∙001 |
| **Ethnicity**  Indo-Caribbean  Afro-Caribbean  Mixed/ others | 460 (41∙7%)  402 (36∙4%)  242 (21∙9%) | 84 (29∙0%)  132 (45∙5%)  74 (25∙5%) | <0∙001 |
| **Ever-smokers** | 302 (27∙4%) | 95 (32∙8%) | 0∙070 |
| **Smoking pack-years**  Never  0-10  10-20  20+ | 802 (72∙7%)  102 (9∙2%)  77 (7∙0%)  121 (11∙0%) | 195 (67∙2%)  39 (13∙4%)  15 (5∙2%)  41 (14∙1%) | 0∙043 |
| **BMI group**^¶^  Underweight  Normal  Overweight  Obesity | 28 (2∙5%)  308 (27∙9%)  378 (34∙2%)  390 (35∙3%) | 12 (4∙1%)  83 (28∙6%)  97 (33∙4%)  98 (33∙8%) | 0∙514 |
| **Doctor diagnosed respiratory disease** | 114 (10∙3%) | 21 (7∙2%) | 0∙114 |
| **Chronic respiratory symptoms** | 382 (34∙6%) | 127 (43∙8%) | 0∙004 |

Data are presented as n (%). BMI = body mass index. ^¶^: Normal BMI = 18.5-25.0 Kg/m^2^; Underweight BMI <18.5 Kg/m^2^; Overweight BMI = 25.0-29.9 Kg/m^2^; Obese BMI ≥30 Kg/m^2^.

TABLE S2. Results of the general linear models analyses for the significant risk factors for Pre-bronchodilator FVC

| **Variables** | **Categories** | **Models with Individual Risk Factors*** | | | **Multivariate Model*** | | | **p-values**  **(Multivariate model)** |
| --- | --- | --- | --- | --- | --- | --- | --- | --- |
| **Coefficient 95% CI Coefficient 95% CI**  **(ml) (ml)** | | | | | | | | |
| **Ethnicity** | Afro-Caribbean  Indo-Caribbean  Mixed/Other | Baseline  -192  98 | -280  -10 | -104  206 | -152  92 | -238  -20 | -66  204 | <0.001 |
| **BMI**^¶^  Kg/m^2^ | Normal  Underweight  Overweight  Obese | Baseline  -120  -38  -166 | -284  -132  -260 | 44  56  -66 | -194  23  -53 | -350  -75  -161 | -38  121  55 | 0.046 |
| **Abnormal waist circumference^§^** | Yes | -249 | -337 | -162 | -190 | -293 | -87 | <0.001 |
| **Abnormal waist–hip ratio** ^Δ^ | Yes | -207 | -288 | -126 | -97 | -172 | -21 | 0.012 |
| **Ever smoked cannabis** | Yes | 207 | 56 | 358 | 179 | 64 | 294 | 0.008 |
| **^Indoor air pollutant exposure^** | None  One  Two  Three | Baseline  -126  -16  -103 | -226  -120  -219 | -26  88  13 | -43  72  -108 | -139  -24  -214 | 53  168  -2 | 0.016 |

| **Education** | None/Primary  Secondary  Vocational  University | Baseline  31  100  280 | -69  -12  148 | 131  212  412 | 23  73  224 | -69  -37  94 | 115  183  354 | 0.046 |
| --- | --- | --- | --- | --- | --- | --- | --- | --- |

*: All models included sex, age, height and height-squared as covariates. ^¶^: Normal BMI = 18.5-25.0 Kg/m^2^; Underweight BMI <18.5 Kg/m^2^; Overweight BMI = 25.0-29.9 Kg/m^2^; Obese BMI ≥30 Kg/m^2^. **^§^:** Abnormal waist circumference ≥ 102 cm for males and ≥ 88 cm for females. ^Δ^: Abnormal wait-hip ratio ≥ 0.90 for males and ≥ 0.85 for females.

TABLE S3. Results of the general linear models analyses for the significant risk factors for Pre-BD FEV1

| **Variables** | **Categories** | **Models with Individual Risk Factors*** | | | **Multivariate Model*** | | | **p-values**  **(Multivariate model)** |
| --- | --- | --- | --- | --- | --- | --- | --- | --- |
| **Coefficient 95% CI Coefficient 95% CI**  **(ml) (ml)** | | | | | | | | |
| **Ethnicity** | Afro-Caribbean  Indo-Caribbean  Mixed/Other | Baseline  -127  72 | -201  -26 | -53  170 | -106  69 | -178  -23 | -34  161 | <0.001 |
| **Abnormal waist circumference^§^** | Yes | -167 | -243 | -91 | -135 | -209 | -61 | <0.001 |
| **Abnormal waist–hip ratio** ^Δ^ | Yes | -137 | -203 | -71 | -69 | -133 | -5 | 0.032 |
| **Indoor air pollutant exposure** | None  One  Two  Three | Baseline  -84  20  -114 | -164  -68  -204 | -4  108  -24 | -35  72  -100 | -115  -12  -186 | 45  156  -14 | 0.005 |

| **Smoking exposure during childhood** | Yes | -63 | -123 | | -3 | -56 | | -114 | | 2 | 0.055 |
| --- | --- | --- | --- | --- | --- | --- | --- | --- | --- | --- | --- |
| **Highest level of education** | None/Primary  Secondary  Vocational  University | Baseline  22  91  226 | | -62  1  116 | 106  181  336 | | -6  40  168 | -82  -54  -50 | 70  134  286 | | 0.032 |

| **Years of education** | 6 years or less  7 years or more | Baseline  109 | 3 | 215 | 92 | -12 | 196 | 0.077 |
| --- | --- | --- | --- | --- | --- | --- | --- | --- |

*: All models included sex, age, height and height-squared as covariates. ^¶^: Normal BMI = 18.5-25.0 Kg/m^2^; Underweight BMI <18.5 Kg/m^2^; Overweight BMI = 25.0-29.9 Kg/m^2^; Obese BMI ≥30 Kg/m^2^. **^§^:** Abnormal waist circumference ≥ 102 cm for males and ≥ 88 cm for females. ^Δ^: Abnormal wait-hip ratio ≥ 0.90 for males and ≥ 0.85 for females

TABLE S4. Results of the general linear models analyses for the significant risk factors for Post-BD FEV1

| **Variables** | **Categories** | **Models with Individual Risk Factors*** | | | **Multivariate Model*** | | | **p-values**  **(Multivariate model)** |
| --- | --- | --- | --- | --- | --- | --- | --- | --- |
| **Coefficient 95% CI Coefficient 95% CI**  **(ml) (ml)** | | | | | | | | |
| **Ethnicity** | Afro-Caribbean  Indo-Caribbean  Mixed/Other | Baseline  -136  63 | -210  -25 | -62  151 | -125  63 | -197  -23 | -53  149 | <0.001 |
| **Abnormal waist circumference^§^** | Yes | -138 | -214 | -61 | -108 | -185 | -32 | 0.005 |
| **Abnormal waist–hip ratio** ^Δ^ | Yes | -127 | -189 | -65 | -56 | -121 | 8 | 0.087 |
| **Indoor air pollutant exposure** | None  One  Two  Three | Baseline  -62  16  -117 | -146  -70  -211 | 22  102  -23 | -20  69  -95 | -100  -11  -183 | 60  149  -7 | 0.009 |

| **Education** | None/Primary  Secondary  Vocational  University | Baseline  15  62  200 | -61  -28  70 | 91  152  330 | 2  30  167 | -70  -56  41 | 74  116  293 | 0.077 |
| --- | --- | --- | --- | --- | --- | --- | --- | --- |

| **Smoking/Symptoms** | Neither  Symptoms  Smoker  Both | Baseline  -109  3  -84 | -185  -87  -206 | -33  93  38 | -85  0  -90 | -157  -86  -202 | -13  86  22 | 0.070 |
| --- | --- | --- | --- | --- | --- | --- | --- | --- |

*: All models included sex, age, height and height-squared as covariates. ^¶^: Normal BMI = 18.5-25.0 Kg/m^2^; Underweight BMI <18.5 Kg/m^2^; Overweight BMI = 25.0-29.9 Kg/m^2^; Obese BMI ≥30 Kg/m^2^. **^§^:** Abnormal waist circumference ≥ 102 cm for males and ≥ 88 cm for females. ^Δ^: Abnormal wait-hip ratio ≥ 0.90 for males and ≥ 0.85 for females.
